# Safety and efficacy evaluation of intracavernosal injection of platelets rich plasma in treatment of vasculogenic erectile dysfunction

**DOI:** 10.1080/20905998.2024.2424459

**Published:** 2024-11-05

**Authors:** Mohammed Abdou Abdel-Rassoul, Amr Amin Mohamed Ragab, Amr Mostafa Ibrahim, Mohamed Abdelwahab, Khaled Mursi Hammoud, Galal Mohamed El Shorbagy

**Affiliations:** Department of Urology, Faculty of Medicine, Cairo University, Cairo, Egypt

**Keywords:** Platelet-rich plasma, erectile dysfunction, vasculogenic, intracavernosal injection

## Abstract

**Objectives:**

The study’s objective is to compare platelet-rich plasma (PRP) injections to placebo in terms of effectiveness and safety for patients with vasculogenic erectile dysfunction (ED).

**Methods:**

This randomized placebo-controlled clinical trial was done on 50 male patients with organic vasculogenic ED and sexually active in a stable heterosexual relationship for a period of over three months. The patients were randomized into two main groups: Group (A) (*n* = 25) underwent PRP treatment. Group (B) (*n* = 25) control underwent placebo treatment.

**Results:**

The two groups under study differed in a statistically significant way regarding the achievement of minimal clinically important differences (MCID) at 3 months and 24 months. Statistically significant variations existed between the mild subgroup and placebo regarding achieving MCID after 3 months and 24 months. The level of satisfaction was statistically significantly higher in the PRP group. A statistically significant variation was present between the mild subgroup and placebo at 3 months and 24 months in terms of changes from baseline in the erectile function domain of the International Index of Erectile Function (IIEF-EF) questionnaire score in each subgroup.

**Conclusions:**

PRP is an effective alternative modality of treatment in cases of ED, and they offer an intermediate stage between pharmaceutical therapy and surgical interventions at least in mild and mild to moderate cases where unsatisfactory results or unpleasant side effects compel the patients to abandon all hope on medical treatment.

## Introduction

Reduced penile blood flow, arterial insufficiency or stenosis, venous leakage, and endothelial dysfunction are the main causes of vasculogenic erectile dysfunction (ED) [1].

Most recommended treatments increase penile hemodynamics to improve erectile function without altering the pathophysiologic causes of ED [2].

ED affects about 52% of the men between the ages of 40 and 70, and predictions indicate that 322 million men worldwide will experience ED symptoms by the year 2025 [3].

Centrifuging whole blood yields the autologous plasma fraction known as platelet-rich plasma (PRP), which has a mean platelet concentration that is 3- to 7-times higher than whole blood [4].

Recent advancements have made PRP intracavernosal injections a promising regenerative and angiogenic treatment option for ED [5].

Animal research suggests that PRP injections may have beneficial anti-inflammatory, reparative, neuroprotective, and neurotrophic properties that may help ameliorate some pathophysiologic mechanisms causing ED [6].

Despite the positive effects of PRP and the rising popularity of regenerative medicine, there is insufficient proof to back up its inclusion in the standard ED therapy algorithm [7].

There is currently an unmet need for high-quality studies investigating the use of PRP for ED treatment due to the dearth of human clinical trials [8].

Previous studies on PRP were limited by a small study group, short follow-up periods, a lack of control groups or groups with placebo, and a lack of quality and quantity analysis of PRP.

In this context, we carried out a randomized, double-blind, placebo-controlled clinical trial to contrast the effectiveness and safety of PRP injections to a control group in patients with vasculogenic ED.

## Materials and methods

Between August 2020 and December 2022, 50 male patients with organic vasculogenic ED and more than three months of sustained heterosexual sexual activity participated in this experimental prospective randomized placebo-controlled clinical trial conducted at Kasr al-Aini Hospital, Cairo University. After approval of the Research Ethics Committee, Faculty of Medicine, Cairo University (MD-194-2021), written informed consent from each patient was taken.

Exclusion criteria included previous major pelvic surgery or trauma, prior major penile surgery or radiation, priapism history, penile fracture, Peyronie’s disease, penile curvature, or any more erectile dysfunction-related anatomical condition, psychogenic ED, history of a severe medical or mental illness that makes it difficult to participate in the study, subjects with partners who reported female sexual dysfunction while conducting the study, or subjects with any other serious health condition. Patients were randomized into two groups: 25 patients in Group (A) who received PRP therapy and 25 control patients with dropout (*n* = 4) in Group (B) who received a placebo. Computer-generated random numbers were used for randomization.

### Preoperative assessment

Pre-treatment, all patients completed the 6 questions of the erectile function domain in the International Index of Erectile Function (IIEF-EF).

### Pretreatment questionnaires

We classified the degrees of ED using the IIEF-EF scoring system.

According to the following: severe (EF score 1 to 10), moderate (EF score 11–16), mild to moderate (EF score of 17 to 21), mild (EF score 22–25), and no ED (EF score 26–30) [9].

People with severe ED (EF score 1–10) who have a change from baseline of seven points or more on the follow-up questionnaire three months after the injection are thought to have minimally clinically important differences (MCID) in the change in the IIEF-EF. Moderate ED (EF score 11–16) is changing from a baseline of five points or more on the follow-up questionnaire at 3 months post-injection. Mild to moderate and mild ED (EF scores 17 to 21 and 22 to 25) have a change from baseline of two points or more on the three-month follow-up questionnaire [10].

We also had a self-developed question on a Likert scale about the patient’s rate of satisfaction after receiving the proposed treatment: Q: On a scale from 0 to 10, how do you rate the level of satisfaction after completion of the treatment protocol? A score of 0 is indicated by extreme dissatisfaction and a score of 10 by extreme satisfaction.

### Imaging

All patients underwent preoperative assessment of the erectile and vascular function using a preoperative colored penile duplex with prostaglandin E1 (alprostadil) intracavernosal injection, with the following data recorded: The erection hardness score (EHS) is a radiologic report that utilizes an objective scale to grade the rigidity of the penis. Grades 1–5 indicate no response, mild tumescence, tumescence without rigidity, partial rigidity suitable for penetration, and total rigidity [11]. The report also measures the diameter of the cavernosal artery before and after the injection of Prostaglandin E1 (PGE1), with normal values defined as an increase of 50–75% from the baseline diameter.

We defined the success in penile duplex parameters as improvement in EHS from baseline after treatment, improvement of PSV that would change the diagnosis of an arteriogenic ED (PSV less than 25 cm/s at 5 min post PGE1 injection), or improvement of EDV parameters that would change the diagnosis of venogenic ED (EDV more than 4 cm/s at 25 min after PGE1 injection) [12].

### Procedure details

We randomly assigned all patients to three sessions, each with a one-week interval of 6–8 mL PRP or normal saline injections. To guarantee allocation concealment and reduce bias in selection, one member of the research team was only in charge of making the injections using the Ycellbio PRP kit (Ycellbio Medical Co. Ltd.) and collecting blood for sampling and administration of injections. We centrifuged the collected blood samples at 4000 rpm for 10 min using a top-loading centrifuge machine and separated the buffy coat. If necessary, we performed a second centrifugation at 4000 rpm for an additional 5 min. PRP was then drawn into a 10-cc syringe and divided into six insulin syringes, each containing 10% calcium chloride for in vitro platelet aggregation cascade activation.

To guarantee the double-blind character of our study, the team member responsible for the injections is different from the one who is doing the penile duplex as well as taking the questionnaires.

Patients were monitored in the clinic for 20–30 min following the surgery to check for any potential complications or side effects.

### Post procedure details

After the treatment regimen concluded, we assessed the participants using the IIEF-EF domain questionnaire at 1, 3, and 24 months, respectively. The penile duplex scan was done after 3 months.

### Sample size calculation

We performed the sample size calculation using G.power 3.1.9.2 (Universitat Kiel, Germany). The sample size was calculated according to the patients with MCID in the IIEF-EF was (76%) in the PRP group and was (25%) in the placebo group according to a previous study [13]. The calculation was based on the following considerations: a 0.05 error and a 95% power of the study. According to the previous calculation based on the previous study, the proper sample size should be 23 patients in each arm. To overcome dropout, we added two cases. Therefore, 25 patients will be allocated in each group.

### Statistical methods

All statistical analyses were performed utilizing IBM SPSS (IBM Corp., Armonk, NY, USA) release 22 for Microsoft Windows. We statistically reported data using mean, standard deviation (SD), median, and range, or frequencies and percentages, as applicable. Utilizing the Kolmogorov–Smirnov test, numerical data were assessed for normal assumptions. The Student t-test was utilized to contrast numerical variables with parametric data, and the Mann-Whitney U test was utilized for non-parametric data. A chi-square test or Fisher’s exact test was utilized to compare categorical data. We used the Spearman rank correlation equation for non-normal variables and the non-linear monotonic relation to determine the correlation between different variables. Statistics were considered significant when the two-sided p-value was less than 0.05.

## Results

**Figure 1.**
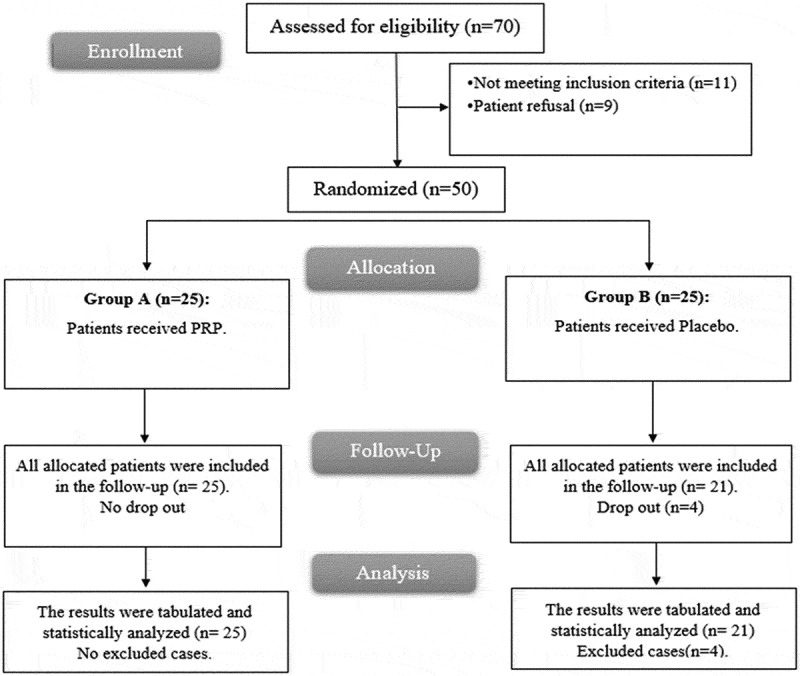
CONSORT flowchart of the of the studied groups.

In this study, 70 patients were assessed for eligibility; 11 patients did not meet the criteria, and 9 patients refused to participate in the study. We randomly allocated the remaining patients into two equal groups, each containing 25 patients. All allocated patients were followed-up analyzed statistically in group A, while 21 allocated patients were followed-up analyzed statistically in group B, and 4 patients dropped out (Figure 1).

There was no statistically significant difference between the two groups in terms of demographics, comorbidities, the IIEF-EF domain questionnaire score before treatment, or the severity of the score on the IIEF-EF domain questionnaire before treatment (Table 1).

**Table 1.** Comparison between the two groups in demographic characteristics, comorbidities, IIEF-EF domain questionnaire pretreatment score, and severity in the IIEF-EF domain questionnaire score before treatment.

	**PRP (n = 25)**	**Placebo (n = 21)**	**p value**
Age	51.1 ± 8.6	47.8 ± 11.3	0.376
BMI	24.8 ± 3.1	23.2 ± 2.2	0.075
DM	13 (52.0%)	8 (38.1%)	0.346
HTN	6 (24.0%)	4 (19.0%)	0.735
CVD	1 (4.0%)	4 (19.0%)	0.163
Smoking	13 (52.0%)	9 (42.9%)	0.536
IIEF-EF Pretreatment	14.4 ± 2.7	15.5 ± 2.8	0.171
Mild ED	9/25 (36.0%)	12/21 (57.1%)	0.328
Moderate ED	15/25 (60.0%)	8/21 (28.1%)	
Severe ED	1/25 (4.0%)	1/21 (4.8%)	

The data are shown as mean ± SD or number (%). *p* > 0.05 = not significant; BMI: body mass index; HTN: hypertension; CVD: cardiovascular diseases; IIEF-EF: Erectile Function domain in the International Index of Erectile Function; ED: erectile dysfunction; PRP: platelet rich plasma.

Between the two research groups, there was a statistically significant variation concerning the achievement of MCID at 3 months and at the end of the clinical trial. With further subgroup analysis, statistically significant variation existed between both arms of the study in patients with mild ED regarding achieving MCID after 3 months and at 24 months, and regarding the moderate ED subgroup, a statistically significant variation was present between both groups at 3 months only and no difference at the end of the trial. A statistically significant variation was detected between PRP treated patients suffering from mild and moderate ED regarding MCID at 3 months and at 24 months (Table 2).

**Table 2.** Percentage of patients achieving MCID in IIEF-EF domain questionnaire in the PRP group (mild and moderate) and placebo and between mild and moderate ED groups treated with PRP.

	**PRP (n = 25)**	**Placebo (n = 21)**	**p value**
3 months	18/25 (72.0%)	5/21 (23.8%)	0.001
24 months	13/25 (52.0%)	4/21 (19.0%)	0.021
Mild ED	**PRP (n = 9)**	**Placebo (n = 12)**	**p value**
3 months	9/9 (100.0%)	5/12 (41.7%)	0.007
24 months	8/9 (88.9%)	4/12 (33.3%)	0.024
Moderate ED	**PRP (n = 15)**	**Placebo (n = 8)**	**p value**
3 months	8/15 (53.3%)	0/8 (0.0%)	0.019
24 months	4/15 (26.7%)	0/8 (0.0%)	0.257
	**PRP mild (n = 9)**	**PRP moderate (n = 15)**	
3 months	9/9 (100%)	8/15 (53.3%)	0.022
24 months	8/9 (88.9%)	4/15 (26.7%)	0.009

MCID: minimal clinically important differences; IIEF-EF: Erectile Function domain in the International Index of Erectile Function; ED: erectile dysfunction; PRP: platelet rich plasma.

The pretreatment IIEF-EF domain questionnaire showed no statistically significant variation between the two groups, but the mild ED subgroup did show a statistically significant variation. In the moderate ED subgroup, there was no statistically significant difference between the two groups when comparing the changes from baseline in the IIEF-EF domain questionnaire score (Table 3).

**Table 3.** The IIEF-EF domain questionnaire in the two groups (PRP and placebo) and in the mild and moderate subgroups.

	**PRP (n = 25)**	**Placebo (n = 21)**	**p value**
Pretreatment	14.4 ± 2.7	15.5 ± 2.8	0.171
1 month	15.7 ± 3.3	16.4 ± 2.8	0.361
3 months	17.8 ± 3.5	16.7 ± 2.8	0.117
24 months	16.8 ± 3.9	15.4 ± 3.2	0.235
Mild ED	**PRP (n = 9)**	**Placebo (n = 12)**	**p value**
Pretreatment	17.3 ± 0.5	17.6 ± 0.8	0.536
1 month	18.9 ± 2.0	18.4 ± 1.4	0.687
3 months	21.0 ± 1.0	18.5 ± 1.4	0.001
24 months	20.6 ± 1.7	17.8 ± 1.8	0.006
Moderate ED	**PRP (n = 15)**	**Placebo (n = 8)**	**p value**
Pretreatment	13.0 ± 1.6	13.0 ± 1.8	0.946
1 month	13.9 ± 2.4	14.0 ± 1.9	0.789
3 months	15.9 ± 3.2	14.3 ± 2.3	0.170
24 months	14.5 ± 3.2	12.4 ± 1.8	0.158

IIEF-EF: Erectile Function domain in the International Index of Erectile Function; ED: erectile dysfunction; PRP: platelet rich plasma.

Between the two groups, there was a statistically significant difference in EDV and EHS, but no statistically significant difference in PSV. On subgroup analysis not statistically, significant variation was found between both (mild and moderate ED) groups and placebo regarding all hemodynamic parameters (PSV, EDV, and EHS), which may be due to the small number of patients in each subgroup. No statistically significant variation existed between the two groups concerning pre-treatment and 3-month EDV and EHS. However, the level of satisfaction was statistically significantly higher in the PRP group (Table 4).

**Table 4.** Comparison between the two groups regarding hemodynamic parameters, and pre-treatment and at 3 months EDV and EHS, and satisfaction.

	**PRP (n = 25)**	**Placebo (n = 21)**	**p value**
PSV Pretreatment	45.3 ± 14.6	43.4 ± 10.8	0.991
PSV 3 months	41.5 ± 9.3	41.1 ± 9.0	0.834
PSV difference	0.0 ± 0.2	0.0 ± 0.2	0.901
EDV difference	0.2 ± 0.4	0.0 ± 0.0	0.032
EHS difference	0.4 ± 0.5	0.1 ± 0.3	0.038
Mild ED	**PRP (n = 9)**	**Placebo (n = 12)**	**p value**
PSV difference	0.0 ± 0.0	0.0 ± 0.0	1.000
EDV difference	0.1 ± 0.3	0.0 ± 0.0	0.248
EHS difference	0.4 ± 0.5	0.2 ± 0.4	0.174
Moderate ED	**PRP (n = 15)**	**Placebo (n = 8)**	**p value**
PSV difference	0.1 ± 0.3	0.1 ± 0.4	0.644
EDV difference	0.2 ± 0.4	0.0 ± 0.0	0.185
EHS difference	0.3 ± 0.5	0.0 ± 0.0	0.116
EDV Pretreatment	3.0 ± 5.2	2.0 ± 3.4	0.655
EDV 3 months	1.2 ± 2.3	2.7 ± 4.0	0.255
EHS Pretreatment	3.2 ± 0.8	3.4 ± 1.0	0.394
EHS 3 months	3.8 ± 0.8	3.4 ± 1.1	0.221
NAD	19 (76.0%)	13 (61.9%)	0.126
Satisfaction	5.9 ± 2.4	4.6 ± 2.0	0.035

PSV: peak systolic velocity; EDV: end diastolic velocity; EHS: Erection hardness score; ED: erectile dysfunction; PRP: platelet rich plasma. NAD: No abnormalities detected.

There was no correlation between risk factors and achieving MCID in the PRP groups at 3 months and at 24 months (Table 5).

**Table 5.** Risk factors and their correlation with achieving MCID in the IIEF-EF domain questionnaire.

	MCID IIEF-EF domain 3 months		MCID IIEF-EF domain 24 months	
	r	p value	r	p value
Age	−0.260	0.209	0.011	0.958
DM	−0.243	0.243	0.038	0.855
HTN	−0.067	0.751	−0.210	0.314
CVD	0.127	0.544	−0.212	0.308
Smoking	0.292	0.156	0.199	0.341
BMI	0.294	0.153	0.372	0.067

MCID: minimal clinically important differences; IIEF-EF: Erectile Function domain in the International Index of Erectile Function; DM: diabetes mellitus; HTN: hypertension; CVD: cardiovascular diseases; BMI: body mass index.

## Discussion

Studies on animals support the hypothesis that PRP injections enhance important aspects of the pathophysiologic pathways causing ED. Platelets were thought to have only hemostatic activity, although in recent years, scientific research and technology have provided a new perspective on platelets and their functions. Studies suggest that platelets contain an abundance of GFs and cytokines that can affect inflammation, angiogenesis, stem cell migration, and cell proliferation [13].

Our study’s objective was to evaluate the safety and effectiveness of intracavernosal injection of PRP in treatment of vasculogenic ED, and considering scarce data and relatively new publications in the literature, we tried to minimize the variables and focus truly on assessing the clinical outcomes of this novel treatment and attain objective results that would shed some light on this new modality of treating vasculogenic ED.

The two groups exhibited a statistically significant difference concerning MCID. Poulios et al. [13] indicated that at three months, 20 out of 29 (69%) patients receiving PRP injections achieved a minimal clinically important difference (MCID) in the IIEF-EF domain questionnaire score, in contrast to 10 out of 26 (39%) patients who received a placebo (*p* = 0.018). Furthermore, we saw a statistically significant difference between the two groups at the conclusion of our clinical study. Poulios et al. [13] indicated that at the 6-month mark, 20 out of 29 (69%) patients receiving PRP injections reported a minimal clinically important difference (MCID) on the IIEF-EF scale, in contrast to 7 out of 26 (27%) patients who received a placebo (*p* < 0.001). In our study, 9 out of 9 patients (100%) in the mild category of the PRP group achieved the Minimal Clinically Important Difference (MCID) in the IIEF-EF domain questionnaire score at 3 months, compared to 5 out of 12 (41%) in the placebo group, with a statistically significant difference (p-value = 0.007). At 24 months, 8 out of 9 patients in the PRP group (89%) achieved MCID versus 4 out of 12 (33%) in the placebo group, also with a statistically significant difference (p-value = 0.024). In the moderate severity category, 8 out of 15 patients (53%) in the PRP group achieved MCID at 3 months, while none (0%) in the placebo group did. At 24 months, only 4 out of 15 patients (26%) in the PRP group maintained their MCID. Poulios and colleagues [13] did not provide data regarding the subgroups.

In this investigation, the alterations from the baseline in the IIEF-EF domain score indicated mean changes of 1.3, 3.4, and 2.4 after 1, 3, and 24 months post-treatment, respectively, with p-values of 0.361, 0.117, and 0.235. Poulios and colleagues [13] documented an enhancement in the IIEF-EF domain score of 2.7 points (*p* = 0.004) at 1 month, 2.8 points (*p* = 0.023) at 3 months, and 3.9 points (*p* < 0.001) at 6 months in patients administered PRP relative to those receiving a placebo.

We observed an EDV difference with a p-value of 0.032 and an EHS variation with a p-value of 0.038, however no difference was noted in PSV.

Concerning pain and safety outcomes (adverse side effects and complications), our study reported no such occurrences, with the exception of one patient who experienced a skin infection and necrosis two months post-PRP injections, which we attributed to his uncontrolled diabetes mellitus. Poulios et al. [13] discovered that participants receiving placebo injections experienced higher mean visual analogue scale (VAS) scores over two sessions compared to those receiving PRP injections (2.6 vs. 2.2, respectively, *p* = 0.008). Neither group exhibited any transient hemorrhagic side effects during the injection or follow-up period, including hematuria, localized petechial bleeding, or ecchymosis.

In this study, we employed a custom-designed questionnaire to assess individuals’ satisfaction with the outcomes. The p-value of 0.035 indicates that the difference in satisfaction rates between the two groups was statistically significant. Poulios and colleagues [13] shown that patients who administered PRP injections expressed greater satisfaction with their treatment outcomes compared to those who received a placebo. The Erectile Dysfunction Inventory of Treatment Satisfaction (EDITS) score following PRP compared to placebo was 62.7 vs 34.5, *p* < 0.001 at 1 month; 62.2 versus 38.5, *p* < 0.001 at 3 months; and 63.2 versus 32.8, *p* < 0.001 at 6 months.

Matz and colleagues [14] indicated that they had only four patients with erectile dysfunction and noted an increase in their IIEF scores; nevertheless, the data was small and lacked substantial informative value. Banno and colleagues [15] indicated that the trial comprised only 9 subjects, with an average IIEF-5 score of 15.6 prior to the injection and a score of 19.9 at 4 weeks post-injection. The score variation lacked statistical significance. Taş and colleagues [16] demonstrated that the mean IIEF-5 score rose from 18 to 20 (*p* < 0.001), although this improvement did not alter the classification of the score as mild IIEF-5 erectile dysfunction (score of 17–21). Bruising occurred as a moderate adverse effect in 8 out of 93 injections administered at the injection site. A fibrotic plaque of 4 mm in diameter developed on the ventral side of the penile shaft of one patient. Alkhayal and Lourdes [17] presented the findings of a study including 267 males with organic, mild-to-moderate erectile dysfunction (ED) who received PRP injections. They also administered the IIEF-5 questionnaire and conducted evaluations at a minimum of six weeks post-therapy. The abstract fails to clarify the fate of the remaining guys or the reasons for their cessation of participation, considering that the investigators possessed complete data on only 61 patients. The mean follow-up duration was about 11 weeks. Prior to treatment, the average IIEF-5 score was 12.5 (indicative of mild-to-moderate erectile dysfunction), while post-treatment, the average IIEF-5 score increased to 17 (indicative of mild erectile dysfunction), *p* < 0.001. No adverse events were observed. Zaghloul and colleagues [18] exhibited a significant enhancement in the IIEF-5 score, with an average increase of 5.5 points. In comparison to the ultrasound examination conducted prior to the PRP injections, there were no changes in the penile duplex hemodynamic parameters. Furthermore, 14 study participants who had previously been unresponsive to PDE5i medications exhibited an enhancement following the administration of these agents. Epifanova and colleagues [19] noted a statistically significant elevation in group 1’s IIEF5 score (*p* > 0.046), peak systolic velocity (PSV) (*p* = 0.005), and resistance index (RI) (*p* = 0.001). In group 2, PSV (*p* = 0.028), RI (*p* = 0.129), and the IIEF5 score (*p* = 0.046) shown improvement. A statistically significant fluctuation was seen in group 3’s IIEF-5 score (*p* < 0.05), but PSV and RI values showed no significant difference (*p* > 0.05). All groups exhibited a significant enhancement in IIEF5 scores throughout the study duration, while disparities were observed across the groups regarding the features of the penile Doppler ultrasound scans.

A thorough review and meta-analysis by Haotian Huang et al. has demonstrated that PRP is significantly more effective and safer in the treatment of ED compared to placebo. The PRP group exhibited markedly elevated IIEF-EF scores. The PRP treatment also favorably influenced peak systolic velocity (PSV) and the minimum clinically meaningful difference (MCID) [20].

Mao, Q. et al. performed a systematic review and meta-analysis of randomized controlled trials from 2024 evaluating the efficacy of platelet-rich plasma in treating erectile dysfunction, concluding that PRP is more effective than placebo, thus offering a promising alternative treatment for ED [21].

A prospective, randomized, double-blind, placebo-controlled clinical trial at the outpatient clinic of the Desai Sethi Urology Institute in Miami, Florida, determined that platelet-rich plasma administered one month apart in men with mild to moderate erectile dysfunction is safe; however, no efficacy difference was noted between platelet-rich plasma and placebo [22].

In our investigation, we aimed to conduct a prospective, placebo-controlled clinical trial with a sufficient patient cohort and an appropriate duration of follow-up. Our objective was to examine the role of risk factors on PRP treatment, along with the alterations in penile duplex hemodynamics that occur as a result of PRP treatment’s effects on cavernous tissue and its impact on patient clinical improvement.

The limitations of our investigation encompass a relatively small patient cohort, a single-center design, and a restricted number of patients with severe erectile dysfunction (ED).

Ultimately, we emphasize the significance of categorizing ED severity into sub-strata. We believe this approach will assist us in identifying the most suitable candidates for this novel treatment of ED and facilitate the development of future nomograms that can precisely forecast the success rate of each patient receiving PRP treatment based on prior data.

## Conclusion

The study results demonstrated significant efficacy and safety of PRP in treating mild to moderate degrees of vasculogenic ED in men at least on the short term with no considerable side effects.

## Data Availability

The data that support the findings of this study are available from the corresponding author upon reasonable request.

## Abbreviations

PRP: platelet-rich plasma
ED: Erectile dysfunction
MCID: minimal clinically important differences
IIEF-EF: International Index of Erectile Function
EHS: erection hardness score
PGE1: Prostaglandin E1
PSV: peak systolic velocity
EDV: end-diastolic velocity
SD: standard deviation
VAS: visual analogue scale
EDITS: The erectile dysfunction inventory of treatment satisfaction
